# Plasma Circulating Tumor DNA Levels for the Monitoring of Melanoma Patients: Landscape of Available Technologies and Clinical Applications

**DOI:** 10.1155/2017/5986129

**Published:** 2017-04-06

**Authors:** Benoit Busser, Julien Lupo, Lucie Sancey, Stéphane Mouret, Patrice Faure, Joel Plumas, Laurence Chaperot, Marie Thérèse Leccia, Jean Luc Coll, Amandine Hurbin, Pierre Hainaut, Julie Charles

**Affiliations:** ^1^Biochemistry Pharmacology and Toxicology Department, Grenoble University Hospital, Grenoble, France; ^2^Institute for Advanced Biosciences, UGA/INSERM U1209/CNRS UMR5309, Grenoble, France; ^3^Virology Laboratory, Grenoble University Hospital, Grenoble, France; ^4^Institut de Biologie Structurale, CEA-CNRS UMR 5075/UGA, Grenoble, France; ^5^Dermatology Unit, Grenoble University Hospital, Grenoble, France; ^6^UGA, Laboratory of Hypoxy Physiopathology Study, INSERM U1042, Grenoble, France; ^7^EFS Rhone-Alpes-Auvergne, Grenoble, France

## Abstract

Melanoma is a cutaneous cancer with an increasing worldwide prevalence and high mortality due to unresectable or metastatic stages. Mutations in* BRAF*,* NRAS*, or* KIT* are present in more than 60% of melanoma cases, but a useful blood-based biomarker for the clinical monitoring of melanoma patients is still lacking. Thus, the analysis of circulating tumor cells (CTCs) and/or cell-free circulating tumor DNA (ctDNA) analysis from blood (liquid biopsies) appears to be a promising noninvasive, repeatable, and systemic sampling tool for detecting and monitoring melanoma. Here, we review the molecular biology-based strategies used for ctDNA quantification in melanoma patients, as well as their main clinical applications. Droplet digital PCR (ddPCR) and next generation sequencing (NGS) technologies appear to be two versatile and complementary strategies to study rare variant mutations for the detection and monitoring of melanoma progression. Among the different clinical uses of ctDNA, we highlight the assessment of molecular heterogeneity and the identification of genetic determinants for targeted therapy as well as the analysis of acquired resistance. Importantly, ctDNA quantification might also be a novel biomarker with a prognostic value for melanoma patients.

## 1. Introduction

It is well established that circulating tumor DNA (ctDNA) is a valid surrogate tumor biomarker for monitoring tumor burden and responses to anticancer therapies [1, 2]. This minimally invasive method to access cancer-derived DNA is also potentially useful for monitoring solid tumors and would avoid the need to perform repeated invasive biopsy procedures [3].

It was recently shown in a prospective proof-of-concept study that the monitoring of metastatic breast cancer via ctDNA was a both highly specific and sensitive strategy [4]. These results even suggest that ctDNA has better sensitivity than the well-established breast tumor biomarker (TM) carbohydrate antigen (CA 15-3). Similarly, serum levels of carcinoembryonic antigen (CEA), CA 19-9, and prostate specific antigen (PSA) can be used as surrogate markers of tumor burden changes in colon, pancreas, and prostate carcinomas. However, there is no valid and specific blood biomarker that is currently used either for the assessment of melanoma burden/recurrence or for clinical monitoring of the disease. The only TM accepted as a standard prognostic factor of survival for melanoma is lactate dehydrogenase (LDH), which is a nonspecific enzyme that can be elevated in various benign or malignant diseases [5].

A relevant blood biomarker for routine clinical melanoma monitoring is thus highly and urgently needed. Melanomas are among the cancers that harbor the highest number of mutations per tumor [6] and ctDNA is detectable in about 80% of cases, with more than 1000 mutant fragments per 5 mL of plasma [7]. Mutations, deletions, or amplifications in* BRAF*,* NRAS*,* TP53*, or* KIT* are generally present in approximately 85% of melanomas [8]. These high-frequency genetic alterations can be found in the blood of melanoma patients and make it possible to distinguish ctDNA from circulating normal DNA. They could collectively serve as specific molecular biomarkers to track ctDNA levels and as personalized biomarkers of melanoma disease.

*BRAF* is the most frequently mutated gene in melanoma [8]. From 43 to 66% of cutaneous melanomas carry* BRAF* mutations, among which the* BRAF* V600E transversion is the most common (80%), followed by V600K (12%), V600R (5%), V600M (4%), and V600D (<5%) [9]. These activating* BRAF* mutations induce the constitutive downstream activation of the MEK-ERK signaling pathway, leading to tumor proliferation and survival [10].

*NRAS* encodes a small GTPase, which was the first protooncogene discovered in melanoma [11], and is found to be mutated in approximately 20% of cases [12]. Both* BRAF* and* NRAS* mutations are predictors of poorer outcome and lower overall survival (OS) of patients than those with nonmutated melanoma [13].

*KIT* is a gene which is found mutated in approximately 3% of melanomas [8]. The positive detection of* KIT* has also been successfully performed in the peripheral blood of patients with gastrointestinal stromal tumors [14, 15]. Patients with melanomas harboring* KIT* mutations are eligible for imatinib therapy [16].

Hence, there is a reason to believe that ctDNA will play the unhoped-for role of the currently missing gold-standard blood-based biomarker for the monitoring of melanoma in the near future.

Here, we review the existing molecular biology approaches that have been used for ctDNA quantification for melanoma patients and describe the main clinical applications and associated results that were obtained.

## 2. Technical Strategies for ctDNA Detection and Quantification

Patients with solid malignancies have higher levels of normal (wild-type) circulating cell-free DNA than healthy individuals [37]. Most conventional PCR-based methods, such as classical Sanger sequencing or pyrosequencing, can detect mutant alleles but are limited by the presence of disproportionate amounts of wild-type alleles in the blood. These two methods can only fulfill the requirements for ctDNA quantification for patients with very high levels of mutant ctDNA, which is rare in plasma [38].

Detecting somatic genetic alterations in the circulation is challenging, but novel approaches have facilitated sensitive and specific detection at low levels. Several recently developed methodologies, such as allele-specific amplification refractory mutation system PCR (ARMS), bead emulsification amplification and magnetics (BEAMing) technology, allele-specific PCR (AS-PCR), droplet digital PCR (ddPCR), and next generation sequencing (NGS), have been used to detect and quantify rare variants in the blood of melanoma patients, with analytical sensitivity ranging from 0.005 to 5% (Table 1).

One of the first studies dedicated to the evaluation of both an innovative ctDNA quantification method and its clinical utility in melanoma showed that quantitative real-time clamp reverse transcription PCR by peptide locked nucleic acid (PNA) and locked nucleic acid hybrid probes (LNAs) detected serum* BRAF* V600E with a sensitivity of 0.001%. The use of allele-specific blockers to suppress the amplification of the wild-type allele was also chosen for the screening of* BRAF* mutant alleles in plasma via competitive allele-specific PCR (CastPCR) [25]. However, the analytical sensitivity was lower at 0.5%.

The detection of* BRAF* mutations in isolated CTCs from* BRAF*-mutated melanoma patients has been performed after an obligatory step of whole genome amplification (WGA) [24]. The authors compared the performance of ddPCR with CastPCR and concluded that ddPCR was more robust and more sensitive than CastPCR. In this study, the detection of* BRAF* mutations with ddPCR reached a sensitivity of 0.0005%, which is 200 times greater than that obtained using CastPCR [24].

AS-PCR methods have recently reached sensitivities below 0.01%, which allow their use for rare variant detection [31].

BEAMing technology is sufficiently sensitive (0.01%, Table 1) to reliably detect mutant ctDNA in the plasma of melanoma patients. For example,* BRAF* mutations were detectable in 76% and 81% of patients with* BRAF* V600E and V600K mutations among 732 patients enrolled in four recent clinical trials evaluating targeted therapies [34].

The sensitivity of AS-PCR is generally not below 0.1% [3, 9, 19, 36]. However, the recent development of a novel, rapid, and inexpensive AS-PCR assay reached a sensitivity of 0.005% due to the use of a PNA designed to inhibit the amplification of the wild-type allele [18].

The technique of ddPCR is a robust method that can detect and quantify very small amounts of ctDNA without the need of a calibration curve [28]. It is indeed more precise for the detection of rare genetic variants and is less sensitive to inhibitors than quantitative RT-PCR [39, 40]. In a recent study, the ddPCR approach reliably distinguished mutant from wild-type alleles with no false positives [29].* BRAF* ctDNA was never detected in a healthy patient cohort, yielding a clinical specificity of 100%. Using the same approach, the* NRAS* ctDNA test showed a specificity higher than 75% [30].

Most of the technologies presented in this review are generally used to detect and quantify known alterations. Despite being useful for longitudinal monitoring, they do not allow the discovery of other mutations, either the other innumerable mutations contained in the tumor or the de novo mutations occurring during the acquisition of resistance mechanisms. From this perspective, sequencing of ctDNA via NGS-based technologies is a reliable strategy that has already succeeded in identifying novel alterations at a frequency as low as one mutant copy in several thousand wild-type copies [41, 42].

Accordingly, digital PCR and NGS technologies are considered to be relevant and complementary diagnostic tools for quantifying rare variants in the blood of cancer patients [40, 43].

Plasma is a better source of ctDNA than serum [19], especially because of the large amounts of wild-type DNA released by white cells lysis during clotting. However, discordant studies have shown that higher levels of ctDNA could be recovered from paired serum samples [9]. There is general agreement concerning the use of either dedicated blood collection tubes that prevent plasma cell-free DNA contamination by cellular DNA [44, 45] or conventional EDTA-containing tubes coupled with a stringent protocol for the separation of the plasma from blood cells by two consecutive centrifugations within 2-3 hours after the blood draw [46].

Analytical sensitivity (Table 1) refers to the estimated fraction of mutated copies that can be detected within the high number of wild-type alleles present in the plasma. The sensitivity of a given method is a percentage that can vary depending on the allele of interest, the technology used, or the pathophysiological state of the patient. For plasma ctDNA quantitation, the use of very sensitive methods is particularly necessary for quantifying the presence of extremely small fractions of mutant alleles (<1.0%). We believe that comparing the absolute sensitivities obtained from various studies aiming to quantify ctDNA is not informative. The diverse experimental protocols used in these studies introduce bias, preventing their comparison. The analysis of ctDNA coming from different sources (plasma, serum, or CTCs), with or without rapid processing after blood collection, obtained using different extraction methods, followed by whole genome amplification or not, and sequenced/quantified using different technologies, impedes any attempt to select the best approach for ctDNA quantification based on claimed (or measured) analytical sensitivities [47]. Importantly, most plasma or serum ctDNA is not derived from CTCs [7].

## 3. Clinical Applications for ctDNA in Melanoma

The recent advances resulting in improved sensitivity and specificity of ctDNA analysis have been shown to be useful in a wide variety of malignancies for several clinical applications, including early cancer detection, assessment of the molecular heterogeneity of overall disease, the monitoring of tumor dynamics, identification of genetic determinants for targeted therapy, evaluation of early treatment responses, monitoring of minimal residual disease, and assessment of the evolution of resistance in real time [38]. Several of these applications have been tested on melanoma patients and the results all support the serious need for routine serial ctDNA monitoring to improve the clinical management of these patients (Table 2).

### 3.1. Assessment of Molecular Heterogeneity

The assessment of genetic heterogeneity is a current technical challenge of major clinical importance. As an example, the mutational status of* BRAF* was found to be different between different sites of the primary tumor (intratumor heterogeneity), between the primary tumor and metastases, and between several metastases from the same patient [21, 48]. Biopsy and subsequent sequencing can miss detecting a mutation because of the site from which it was taken. A potential strategy to overcome such a bias in routine sequencing is to sequence plasma ctDNA. Indeed, ctDNA is a valid source for theoretically finding all mutations present in all tumor sites of a given patient [38].

### 3.2. Prognostic Value

The highest levels of circulating* BRAF* V600E were found in more advanced stages of disease [17]. This finding was in accordance with results of another seminal study, dedicated to quantifying circulating* BRAF* mutations in the blood of melanoma patients, which showed that detectable* BRAF* levels were only found in advanced stage III/IV disease [9]. The authors of this study postulated that this blood biomarker would not represent an interesting early-stage marker for melanoma patients, despite the fact that* BRAF* mutations occur early in melanomagenesis [49].

The prognostic significance of ctDNA quantification in melanoma has more recently been clearly demonstrated. Patients with OS of more than two years had significantly fewer copies of mutant* BRAF* V600E per mL of plasma than patients with OS of less than two years [28]. The prognostic value of ctDNA levels was also demonstrated in a phase II cohort of melanoma patients which included histologically confirmed patients with* BRAF* mutant stage IV melanoma [32]. Increasing amounts of circulating* BRAF* V600E were associated with a reduced overall response rate and shorter progression-free survival (PFS). Elevated ctDNA levels were also significantly associated with both shorter PFS and lower OS in uveal melanoma [26]. In addition, the absence of detectable ctDNA prior to treatment (baseline) within a large cohort of melanoma patients with* BRAF* mutations was an independent predictor of better disease outcome. Patients negative for* BRAF* ctDNA had higher response rates to dabrafenib and trametinib, longer PFS, and higher OS than patients for whom* BRAF* mutations were detected in the plasma [34]. Longer PFS also correlated with undetectable ctDNA levels after treatment initiation in an independent 36-patient cohort receiving dabrafenib and trametinib [31]. In a 48-patient cohort treated by MAPK inhibitors or immunotherapies, low baseline ctDNA was a good predictor of response to treatment and longer PFS [30]. More recently, the presence of detectable levels of ctDNA at baseline strongly correlated with longer OS [20]. All these studies suggest that rising or elevated ctDNA levels may predict poorer clinical outcome.

### 3.3. Identification of Genetic Determinants for Targeted Therapy

A phase II study of a large cohort of* BRAF* mutant melanoma patients failed to demonstrate the prognostic value of ctDNA detection in advanced melanoma. However, the authors stressed that there was a strong rationale for using blood testing to identify whether there are any genetic determinants for associated targeted therapy [3]. The definitive evidence of the benefit of using plasma rather than serum for ctDNA studies was provided in a large cohort of melanoma patients [19]. In this study, the authors proposed that* BRAF* V600 mutation testing from ctDNA should be considered as a first screening test for positively selecting patients with* BRAF* mutations eligible for targeted therapy. This blood-based mutation screening approach could significantly shorten the turnaround time relative to that required for mutation testing from archived FFPE tumors [19].

### 3.4. Monitoring of the Disease

The first attempt to monitor ctDNA levels in melanoma patients before and after treatment resulted in a significant positive correlation between the level of remaining detectable serum* BRAF* V600E and the absence of response to bio/chemotherapy [17]. Another study reported a rapid and marked decrease in* BRAF* V600E blood levels following the initiation of treatment, whether it was surgical or based on targeted anti-BRAF and/or anti-MEK therapies. The decrease correlated with the response to treatment, shown by imaging [22].

The longitudinal variations of ctDNA levels during the treatment of melanoma are logarithmic [28], reinforcing its potential role as a relevant surrogate marker for monitoring efficacy (decrease and/or minimal residual disease) and recurrence (increase after nadir) of melanoma therapy. The only blood TM routinely incorporated in the management of melanoma is LDH, but it is neither sensitive nor specific and is considered to be an unreliable marker for monitoring treatment response [29]. A small case series demonstrated that ddPCR-based ctDNA quantification allowed the monitoring of treatment responses or the emergence of tumor resistance more consistently and informatively than LDH [29]. The superiority of measuring ctDNA over that of LDH levels was demonstrated in three other patients for whom ctDNA levels more accurately reflected the evolution of disease than those of LDH, as the latter tends to increase following immunotherapy [35]. In contrast, two other studies reported a positive correlation between ctDNA and LDH levels [20, 30].

Changes in ctDNA levels that occurred in patients receiving immune checkpoint blocking agents were predictive of treatment efficacy [33]. In this study, the authors also postulated that (1) decreases in ctDNA levels could help to identify patients who are responding to treatment, (2) increases in ctDNA levels might indicate tumor progression or tumor lysis preceding regression, (3) rising ctDNA levels after a long period off treatment might reflect tumor recurrence, and (4) ctDNA could be used as a marker of minimal residual disease after surgical resection. The monitoring of metastatic melanoma patients receiving targeted therapies also suggested that ctDNA levels dramatically decline during the first weeks of therapy, sometimes decreasing to undetectable levels, which is also associated with response to therapy [30]. An increase of ctDNA levels was never found in patients with an ongoing response but was found in 19/27 patients with progressive disease under targeted therapy, thus resulting in a sensitivity of 70% and a specificity of 100% [31].

The interest of such a longitudinal follow-up is that the recurrence of tumor burden was apparent by ctDNA analysis when radiological analysis still classified the tumor as stable [30]. That biological recurrence precedes radiological signs of recurrence by several weeks is well established [50, 51].

### 3.5. Resistance to Treatment

The assessment of tumor resistance to a given treatment can be determined by ctDNA studies in a dual manner. The first option consists of detecting increased levels of ctDNA carrying the original mutation after a prolonged period of undetectable or low values.* BRAF* mutations are usually conserved when recurrence/resistance to targeted therapies occurs and concentrations of plasma ctDNA generally decrease after treatment initiation [30]. Therefore, if progressive disease occurs after a period of tumor response, it is likely that an increase of ctDNA levels with the same primary* BRAF* mutation as that of the relapsing tumor cells will be detected. The second option consists of detecting resistance mutations. For melanoma treated with BRAF inhibitors, resistance generally occurs within the first year and usually involves reactivation of the MAPK pathway by the acquisition of secondary* NRAS* or* MEK* mutations or alternative splicing or amplification of the* BRAF* gene itself [52]. Tracking such secondary mutations is an approach that has already demonstrated its utility in the routine clinical monitoring for the early recurrence of lung cancers [53]. For melanoma patients, ctDNA monitoring of seven patients with progressive disease after a prior response to anti-BRAF treatments allowed the identification of secondary resistance mutations of the* NRAS* gene for three of them [30]. Similarly, a retrospective analysis of ctDNA from patients with tumor resistance was also performed by whole exome sequencing (WES) [35] and identified secondary* NRAS* mutations. A specific 10-gene panel was further developed and successfully used for the assessment in plasma of secondary mutations known to mediate resistance to BRAF and MEK inhibitors [35].

## 4. General Conclusion

Liquid biopsies are a promising area of investigation for improving the clinical monitoring of patients with solid malignancies [7] and ctDNA has been demonstrated for several applications to be a diagnostic, prognostic, and predictive biomarker for several malignancies [47]. The recent progress in the analytical sensitivity of digital genomic technologies allows the investigation of very rare mutant variants of ctDNA, representing an indisputable advantage over most strategies requiring CTC isolation [7]. The routine sequencing of* BRAF* (exon 15),* NRAS* (exon 2), and* KIT* (exons 9, 11, 13, and 17) for clinical purposes, such as molecular-based targeted therapy, suggests that tailored monitoring of the kinetics of plasma ctDNA could be used in the near future for melanoma patients for whom a mutation is detected.

Indeed, ctDNA can also harbor epigenetic modifications, such as methylation that has already been identified as a relevant prognostic factor for melanoma patients [54, 55]. Digital PCR and NGS strategies appear to be two of the most versatile technologies to study rare variants, such as mutations for cancer or SNPs for pharmacogenetics, as well as methylation [56].

Given the heterogeneity of tumor mutations between primary and metastatic sites, or between several metastatic sites, ctDNA might provide a more relevant representation of the mutational status of a patient than a biopsy from a single lesion or site [3, 20, 28]. Liquid biopsies, especially plasma ctDNA, have made the analysis of acquired resistance to anticancer treatments possible [50]. Recent studies showed that the monitoring of plasma ctDNA accurately reflected real-time sampling of multifocal tumor evolution [57]. These findings are fully transposable to melanoma patients, and such monitoring will help clinicians to better understand the molecular evolution of the individual primary and metastatic tumors of their patients. Further studies on large cohorts will be needed to prospectively evaluate the benefit of using serial ctDNA investigations in the monitoring of melanoma patients.

## Figures and Tables

**Table 1:** Overview of techniques used for detection and quantification of plasma ctDNA for melanoma patients. PCR: polymerase chain reaction; AS-PCR: allele-specific PCR; ARMS: amplification refractory mutation system allele-specific PCR; MS-PCR: mutant-specific PCR with fluorescent detection; CastPCR: competitive allele-specific PCR; CTCs: circulating tumor cells; Bi-PAP: mutation-specific bidirectional pyrophosphorolysis-activated polymerization; ddPCR: droplet digital PCR; BEAMing: beads, emulsification, amplification, and magnetics; NGS: next generation sequencing; WES: whole exome sequencing.

Method	Gene (mutation)	Analytical sensitivity (% of mutated copies)	References
Quantitative real-time clamp reverse transcription PCR	*BRAF* (p.V600E)	0.001%	[17]

AS-PCR or ARMS	*BRAF* (p.V600E/K/D)	0.1%	[3]

AS-PCR or ARMS	*BRAF* (p.V600E)	0.3%	[18]

AS-PCR or ARMS	*BRAF* (p.V600E)	0.25%	[9]

AS-PCR or ARMS	*BRAF* (p.V600E)	2.0%	[19]

ARMS	*BRAF* (p.V600E) *BRAF* (p.V600D) *BRAF* (p.V600K) *BRAF* (p.V600R)	1.82% 3.19% 4.34% 4.85%	[20]

MS-PCR	*BRAF* (p.V600E)	0.01%	[21]

RT-PCR + restriction enzyme digestion	*BRAF* (p.V600E)	0.1%	[22, 23]

ddPCR on DNA from CTC	*BRAF* (p.V600E) *BRAF* (p.V600K)	0.0005% after WGA enrichment	[24]

CastPCR on DNA from CTC	*BRAF* (p.V600E) *BRAF* (p.V600K)	0.1% after WGA enrichment	[24]

CastPCR	*BRAF* (p.V600E)	0.5%	[25]

Bi-PAP	*GNAQ* (c.626A>T) *GNAQ* (c.626A>C) *GNA11* (c.626A>T)	~0.05%	[26, 27]

ddPCR	*BRAF* (p.V600E)	0.005%	[28]

ddPCR	*BRAF* (p.V600E) *BRAF* (p.V600K) *NRAS* (p.Q61H)	0.01%	[29]

ddPCR	*BRAF* (p.V600E) *BRAF* (p.V600K) *NRAS* (p.Q61K) *NRAS* (p.Q61R)	0.01%	[30]

AS-PCR or ARMS	*BRAF* (p.V600E)	0.005%	[18]

AS-PCR	*BRAF* (p.V600E/E2/D/K/R/M)	0.01%	[31]

BEAMing technology	*BRAF* (p.V600E) *BRAF* (p.V600K)		[32]

BEAMing technology	*BRAF* (p.V600E) *NRAS* (p.Q61K) *NRAS* (p.Q61R)	<0.01%	[33]

BEAMing technology	*BRAF* (p.V600E) *BRAF* (p.V600K)	0.01%	[34]

PCR+NGS	*TERT* promoter	<0.1%	[33]

NGS (WES)	Exome		[35]

**Table 2:** Applications of ctDNA quantification and monitoring for melanoma patients. PBL: peripheral blood lymphocytes.

Gene	Sample	Application	References
*BRAF*	Serum	(i) Tumor response monitoring (ii) Prognostic value	[17]

*BRAF*	Serum/plasma	Advanced stage IV monitoring	[9]

*BRAF*	Serum	Identification of genetic determinants for targeted therapy	[3]

*BRAF*	Plasma	Identification of genetic determinants for targeted therapy	[21]

*BRAF*	Serum/plasma	Identification of genetic determinants for targeted therapy	[19]

*BRAF*	Plasma	Identification of genetic determinants for targeted therapy	[36]

*BRAF*	PBL	(i) Identification of genetic determinants for targeted therapy (ii) Evaluation of early treatment response (iii) Monitoring of minimal residual disease	[22]

*GNAQ* *GNA11*	Plasma	Prognostic value	[26]

*BRAF*	Plasma	(i) Identification of genetic determinants for targeted therapy (ii) Monitoring of tumor dynamics (iii) Prognostic value	[28]

*BRAF* *NRAS* *TERT* Promoter	Plasma	Evaluation of early treatment response to immunotherapy	[33]

*BRAF*	Plasma/serum	(i) Prognostic value (ii) Monitoring of tumor dynamics (iii) Evaluation of early treatment response (iv) Monitoring of minimal residual disease	[18]

*BRAF*	Plasma	Prognostic value	[32]

*BRAF* *NRAS*	Plasma	(i) Monitoring of tumor dynamics (ii) Monitoring of minimal residual disease (iii) Real-time assessment of resistance	[30]

*BRAF*	Plasma	Prognostic value	[34]

*BRAF*	Plasma	(i) Prognostic value (ii) Monitoring of tumor dynamics (iii) Evaluation of early treatment response (iv) Real-time assessment of resistance	[34]

*BRAF* *NRAS*	Plasma	(i) Prognostic value (ii) Evaluation of early treatment response (iii) Real-time assessment of resistance	[30]

*BRAF*	Plasma	(i) Identification of genetic determinants for targeted therapy (ii) Prognostic value	[20]

Exome	Plasma	(i) Evaluation of early treatment response (ii) Real-time assessment of resistance	[35]
